# Four Loci Explain 83% of Size Variation in the Horse

**DOI:** 10.1371/journal.pone.0039929

**Published:** 2012-07-11

**Authors:** Shokouh Makvandi-Nejad, Gabriel E. Hoffman, Jeremy J. Allen, Erin Chu, Esther Gu, Alyssa M. Chandler, Ariel I. Loredo, Rebecca R. Bellone, Jason G. Mezey, Samantha A. Brooks, Nathan B. Sutter

**Affiliations:** 1 Department of Clinical Sciences, Cornell University, Ithaca, New York, United States of America; 2 Department of Biological Statistics and Computational Biology, Cornell University, Ithaca, New York, United States of America; 3 Biology Department, La Sierra University, Riverside, California, United States of America; 4 Department of Biology, University of Tampa, Tampa, Florida, United States of America; 5 Department of Animal Science, Cornell University, Ithaca, New York, United States of America; University of York, United Kingdom

## Abstract

Horse body size varies greatly due to intense selection within each breed. American Miniatures are less than one meter tall at the withers while Shires and Percherons can exceed two meters. The genetic basis for this variation is not known. We hypothesize that the breed population structure of the horse should simplify efforts to identify genes controlling size. In support of this, here we show with genome-wide association scans (GWAS) that genetic variation at just four loci can explain the great majority of horse size variation. Unlike humans, which are naturally reproducing and possess many genetic variants with weak effects on size, we show that horses, like other domestic mammals, carry just a small number of size loci with alleles of large effect. Furthermore, three of our horse size loci contain the *LCORL*, *HMGA2* and *ZFAT* genes that have previously been found to control human height. The *LCORL/NCAPG* locus is also implicated in cattle growth and *HMGA2* is associated with dog size. Extreme size diversification is a hallmark of domestication. Our results in the horse, complemented by the prior work in cattle and dog, serve to pinpoint those very few genes that have played major roles in the rapid evolution of size during domestication.

## Introduction

The horse, like other domestic mammals, is comprised of many inbred and highly selected breed populations. Like all domestic mammals, the horse has experienced intense selection for certain traits. For example, extreme size diversification is a hallmark of domestication [1] and horses are no exception to this pattern. Today, horse breeds like the American Miniature average less than one meter tall at the withers while Shires and Percherons can exceed two meters [2]. The genetic basis for horse size variation is not known but we hypothesize that the breed population structure of the horse should simplify efforts to identify genes controlling size.

Size is a highly complex trait and until recently no human variants contributing to natural size variation had been found. Now, genome-wide association scans (GWAS) and meta-analyses with large sample sizes have identified nearly 200 size loci in the human genome [3]–[14]. Control of human size is mediated by a huge number of genes of very small effect [15]. In fact, it has been estimated that 697 genes, if identified, would explain just 15.7% of variance in human height [3]. In contrast, a single gene, *IGF1*, explains ∼10–15% of dog size variation [16], [17] and the majority of dog breed-average mass can be explained by as few as six loci [18]. Domestic mammals therefore offer a powerful system in which to investigate genes controlling size. In support of this, here we show with two GWAS that genetic variation at just four loci can explain the great majority of horse size variation. Unlike humans, which are naturally reproducing and possess many genetic variants with weak effects on size [3], [15], we show that horses, like other domestic mammals [18]–[20], carry just a small number of size loci with alleles of large effect.

## Results and Discussion

With the ultimate goal of understanding the genetics of size and the rapid changes in size that have occurred in species under domestication, we previously quantified horse size variation by collecting 33 measurements of the head, neck, trunk and limbs from each of 1215 horses of known breed [2]. Our principal components (PC) analysis of the measurements showed that PC1 (which we will refer to as ‘PC1-size’) quantifies overall horse size and explains 65.9% of the variance in the body measurements [2].

**Figure 1:**
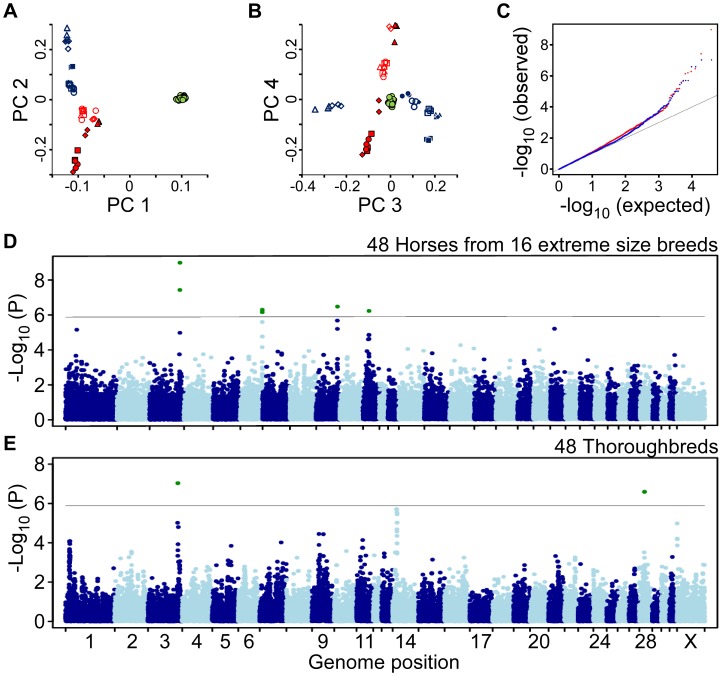
Two genome-wide association scans for size identify five significantly associated loci. (A) Horse breed phylogenetic relationships were inferred using a principal components analysis of SNP genotypes collected from 48 horses from 16 extreme size breeds plus 48 Thoroughbreds. Thoroughbreds (filled green circles) and other breeds have distinct genetic signatures. Large breeds (blue points) are genetically clustered in PC-space as are small breeds (red points). The first four PCs are plotted: PC1 (9.7% of variance explained) and PC2 (2.7%) and in (B) PC3 (2.4%) and PC4 (2.0%). Breeds in size order: American Miniature  =  filled red squares, Falabella  =  filled red circles, Caspian  =  filled red triangles, Shetland Pony  =  filled red diamonds, Welsh Mountain Pony  =  unfilled red squares, Welsh Pony  =  unfilled red circles, Dartmoor Pony  =  unfilled red triangles, Puerto Rican Paso Fino  =  unfilled red diamonds, Friesian  =  filled blue squares, Suffolk Punch  =  filled blue circles, Ardennais  =  filled blue triangles, Brabant  =  filled blue diamonds, Belgian  =  unfilled blue squares, Percheron  =  unfilled blue circles, Clydesdale  =  unfilled blue triangles, and Shire  =  unfilled blue diamonds. (C) Quantile-quantile plot for the GWA scans. The p-values for the 16 breed scan are plotted in red and have a genomic inflation factor of 1.189. The p-values for the Thoroughbred scan are plotted in blue and have a genomic inflation factor of 1.114. (D) Manhattan plot for the GWA scan of 48 horses from 16 breeds of extreme size and (E) 48 Thoroughbreds. The horizontal line in each indicates genome-wide significance with an alpha  = 0.05 and Bonferroni correction for multiple hypothesis testing.

To identify genes controlling PC1-size variation we conducted two GWAS (Fig. 1) using the equine 50 K SNP genotyping chip (Illumina, Inc.). DNA was collected from 48 horses of 16 different large and small breeds (three horses per breed) plus 48 Thoroughbreds of variable size. We inspected pedigrees to avoid including close relatives. The equine 50 K SNP chip has a ∼5 Mbp gap in coverage on chromosome 6. Because *high mobility group AT-hook 2 (HMGA2)* is within this interval and is a strong candidate for size [3]–[10], [12], [13], we added SNP genotypes from the *HMGA2* locus to both GWA scans. We discovered and genotyped 34 SNPs in and around *HMGA2* using two-direction capillary sequencing of seven amplicons in each of our 96 horse samples.

We first examined the genotypes via a principal components analysis to assess breed phylogenetic relationships (Fig. 1A–B). Each breed has a distinct genetic signature, as was found in a recent horse phylogeny [21]. The PC1 axis of variation distinguishes between the thoroughbreds and all the other breeds in our sample, i.e. the 16 breeds of extreme size. It makes sense that these SNPs would readily distinguish the thoroughbred breed, because a thoroughbred’s genome was sequenced to provide the horse reference. As a consequence, a disproportionate number of the total SNP discoveries in the horse species have involved sequences from thoroughbred chromosomes. Interestingly, we find that breeds assort on the genotype PC2, PC3 and PC4 axes largely by size (Fig. 1A–B). The PC2 axis separates our eight sampled large breeds from our eight small breeds. Furthermore, the PC3 axis separates the very largest breeds (Shire and Clydesdale) from the other large breeds, and PC4 separates three of the smallest breeds (American Miniature, Falabella and Shetland Pony) from the other small breeds. This finding supports a model of horse evolution in which divergence and genetic differentiation according to body size occurred early and was subsequently followed by creation of breed lines. The GWAS were conducted using EMMA [22] to correct for population structure, with sex included as a covariate. Markers with <10% minor allele frequency or >20% missing genotypes were excluded. No samples were excluded. Following EMMA correction, the GWA scans using 16 horse breeds and Thoroughbreds had genomic inflation factors [23] of 1.189 and 1.114, respectively (Fig. 1C).

The 16 breed GWAS was conducted with 48 of our measured horses that have extreme PC1-size values. We selected three horses from each of eight small and eight large breeds (Fig. 1D). In the Thoroughbred GWAS we genotyped 24 small and 24 large Thoroughbred horses, which represent the ∼10% smallest and ∼10% largest horses for PC1-size among the 219 Thoroughbreds we measured (Fig. 1E). This multi-breed design tests our hypothesis that many of the alleles controlling size are likely to be shared widely across extreme-sized breeds and in some cases, may contribute to size variation within breeds. Limited locus and allelic heterogeneity, and breed sharing of alleles identical-by-descent, is a common pattern for traits under selection in domestic mammals [16], [18], [21], [24]–[26].

**Figure 2:**
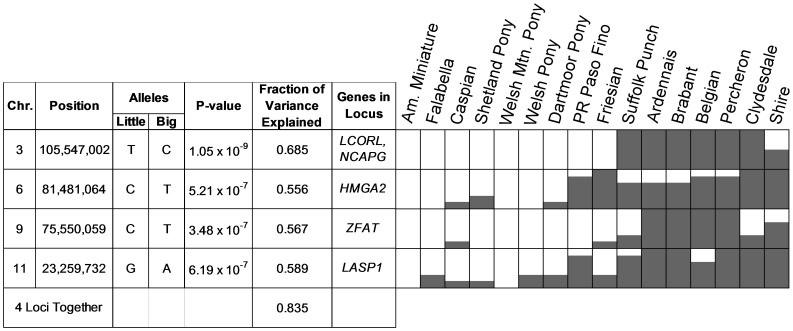
The 16 breed GWA scan identifies four loci associated with horse size. On the right the breeds are shown in size order. For each locus and breed the dark column in the cell indicates the frequency of the allele that is associated with large size.

We have identified four loci in the 16 breed scan and two loci in the Thoroughbred scan that are significantly associated with horse size following Bonferroni correction for multiple hypothesis testing (Fig. 1D–E and Fig. 2). The locus on chromosome 3 was identified independently in both scans. The four loci on chromosomes 3, 6, 9 and 11 together explain 83% of size variance in the 48 horses from 16 breeds (Fig. 2). Together, the loci on chromosomes 3 and 28 explain an estimated 59% of the variance in Thoroughbred size. While these estimates are likely to be upwardly biased by our small sample size, they nevertheless make the qualitative point that the genetic control of horse size includes loci with large effects. The simplicity of the genetic control of horse size contrasts greatly with the complexity of human size genetics [3], [15] but is similar to results for the domestic dog [18], [20].

**Figure 3:**
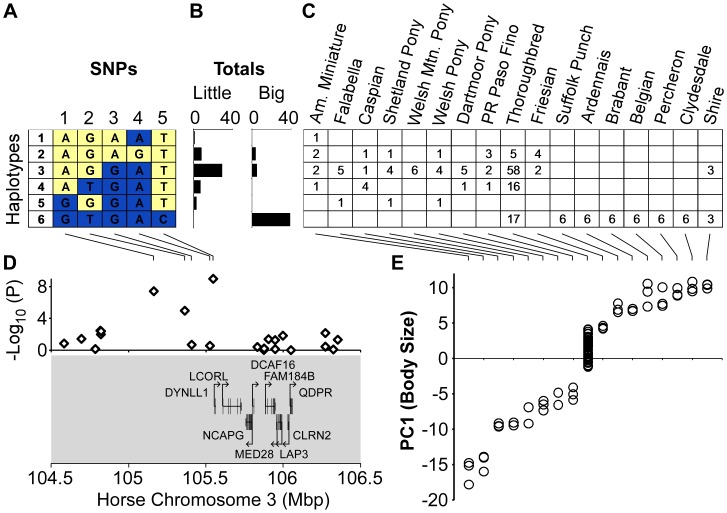
Haplotypes at the *LCORL* locus on horse chromosome 3 are associated with size. (A) Haplotypes for five SNPs were inferred with PHASE and (B) have different counts in the set of all little breed horses (American Miniature to Puerto Rican Paso Fino) vs. all big breed horses (Friesian to Shire). (C) For each breed, the count of chromosomes inferred to be carrying each haplotype. (D) Size-associated SNPs on chromosome 3 are adjacent to the *LCORL* gene. SNP associations from the 16-breed GWA scan are plotted above the genes in this locus. Each gene’s exons (vertical bars), introns (horizontal lines) and direction of transcription (arrow) are indicated. (E) The size of each horse was quantified via a principal components analysis of 33 measurements from the head, neck, trunk and limbs. For each horse, breed membership is plotted vs. PC1-size score.

The top genome-wide associated SNP in both GWAS is on chromosome 3 at 105,547,002 bp and is located 100 kb upstream of the *ligand dependent nuclear receptor corepressor-like (LCORL)* gene. The association signal at this SNP is near its maximum possible value in our 16 breed scan, as the alleles nearly perfectly segregate by size (Fig. 3). The *LCORL* gene is a transcription factor that has repeatedly been associated with human height 3,5,6,8–14. In cattle *LCORL* was identified in a screen for loci under selection [27] and the immediately adjacent gene, *NCAPG*, has been implicated in prenatal growth [28]. We inferred haplotypes for SNPs flanking the associated SNP (Fig. 3A–D). Haplotype #3 is found in all eight small breeds but only two large breeds (Fig. 3C). Together the eight small breeds carry five different haplotypes. In contrast, haplotype diversity is low in the large breeds, as six of them carry just a single haplotype, consistent with a selective sweep at this locus. The sizes of individual horses are plotted in Fig. 3E.

**Figure 4:**
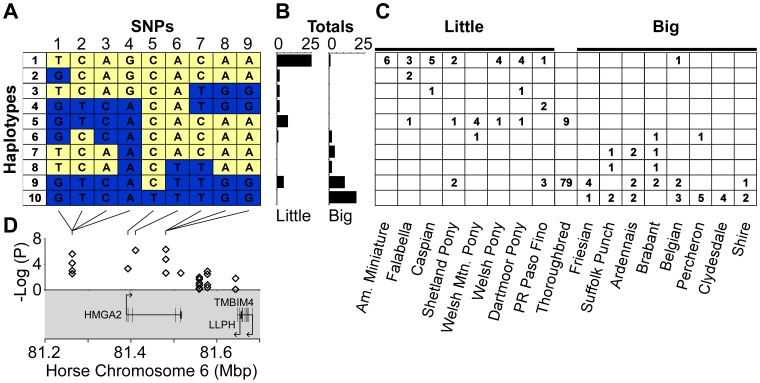
Association with size at the *HMGA2* locus. (A) Haplotypes spanning *HMGA2* were inferred with PHASE for a set of nine SNPs that included all size-associated SNPs at the locus. (B) The haplotype counts differ in the set of all little breed horses (American Miniature to Puerto Rican Paso Fino) vs. all big breed horses (Friesian to Shire). (C) For each breed, the count of chromosomes inferred to be carrying that haplotype. (D) Size-associated SNPs occur within and adjacent to *HMGA2*. SNP associations from the 16-breed GWA scan are plotted above the genes in this locus. These SNPs were discovered and genotyped by capillary sequencing (see main text). Each gene’s exons (vertical bars), introns (horizontal lines) and direction of transcription (arrow) are indicated.

We also found a significant association with horse size for SNPs within and adjacent to *HMGA2* (Fig. 4). We inferred 9-SNP haplotypes and found 10 haplotypes above a 1% frequency (Fig. 4A). Haplotype #1 is carried on 55% of the little horse chromosomes but just a single large horse chromosome (Fig. 4B, C). Haplotype #10, in contrast, is common in large breeds but not found in any small breeds (Fig. 4C). *HMGA2* is an architectural transcription factor that regulates gene expression and directs cellular growth, proliferation and differentiation [29]. It was the first gene in which a common variant was associated with human height [4] and this finding has been replicated in many different human populations [3], [5], [7]–[10], [12], [13]. Mice homozygous for a *HMGA2* knockout are just 40% the body weight of controls [30]. Furthermore, the *HMGA2* locus has twice been associated with size in dogs [18], [20].

Our association on chromosome 9 is intergenic in a gene-sparse region 410 kbp upstream of the transcription factor [31] *zinc finger and AT hook domain containing (ZFAT)*, which has been associated with height in multiple human populations [3], [11], [12]. ZFAT plays a role during development in hematopoiesis and mice homozygous for a knockout of the gene die as embryos [31].

For the other statistically associated SNPs, the association in the 16 breed scan on chromosome 11 is in the first intron of the *LIM and SH3 protein 1 (LASP1)* gene, which occurs in a gene-rich region. LASP1 mediates cell migration and survival and its expression is induced by IGF1 [32]. Its mis-expression in the mouse disrupts chondrocyte differentiation [33]. Thus, *LASP1* is a good candidate for further investigation. However, the locus is gene-dense and fine-mapping will be needed to identify the causal variant or variants contributing to size variation. The Thoroughbred association on chromosome 28 is at a pair of SNPs 3 kbp apart at 18,161,215 bp and 18,164,558 bp. The SNPs are in perfect linkage disequilibrium and are intergenic between *chronic lymphocytic leukemia up-regulated 1* (*CLLU1)* and *plekstrin homology domain containing, family G member 7* (*PLEKHG7)*. The 16 breed scan does not show any association with size at this locus (Fig. 1D), so genotyping in additional Thoroughbreds will be the best way to confirm and refine the association. On chromosome 14 the Thoroughbred scan identified a marginally significant association (Fig. 1E) for a set of SNPs spanning a large interval from 14.7–16.4 Mbp. This region in the horse reference genome assembly lacks genes except for a pair of pseudogenes. One of the pseudogenes is derived from *vacuolar protein sorting 4 homology A* (*VPS4A*), the protein product of which was recently shown to interact with Ras to promote growth factor signaling [34].

Three of the five significant loci we identified have previously been associated with size in humans, which argues against them being false positives. This finding also illustrates the conservation of size determination in mammals and makes possible a comparison of the evolution of these genes in natural versus intensely selected species.

Nearly 1% of all human genes are now implicated in contributing to size variation [3]. We show here that, in stark contrast, the control of the majority of horse size is genetically fairly simple. Genes controlling size in the horse are drawn largely from the broad set already identified in this role in humans. By combining our results with previous findings in cattle and dog we have identified a very short list of genes that were selected repeatedly in domestication to act as major drivers of rapid and extreme size diversification. We hypothesize that *HMGA2* or *LCORL*, or both, may also drive size variation in other domestic mammals. By highlighting here a small but important subset of the size genes found in humans, the horse also offers guidance for exploring size genetics in humans and other mammals.

Note added in proof: while this paper was under review, complementary data describing genome-wide associations with withers height for the *LCORL*/*NCAPG* and *ZFAT* loci were reported for Franches-Montagnes horses [35].

## Materials and Methods

### Ethics Statement

Horses were sampled with signed consent from owners under a protocol approved by the institutional animal care and use committee at Cornell University.

### Sample Collection and Phenotyping

A total of 33 measurements, breed identity, sex and date of birth were collected for each horse, as previously described [2]. Pedigrees and photographs were also collected and were used to confirm owner statements of breed identity. Pedigrees were also inspected to avoid genotyping close relatives. DNA was extracted from tail hair bulbs or blood using standard methods. The measurement data from a total of 1215 horses representing 65 breeds were subjected to a correlation matrix principal components analysis (R; princomp() function) to quantify PC1-size for each horse. See ref. [2] for details.

### Genotyping and Genome-wide Association Analysis

Genome-wide SNP genotypes were collected for 96 horses using the equine 50 K SNP chip (Illumina, Inc.). The 16 breed sample and the Thoroughbred sample were each run as their own batches at Geneseek, Inc. The Illumina software genotype calls were used. SNPs were removed from the analysis if more than 20% of the samples had a missing genotype or if the minor allele frequency was less than 10%. No samples were removed from the analysis. After filtering, 48 samples and 37,584 SNPs were analyzed in the 16 breed GWA scan, and 48 samples and 38,496 SNPs were analyzed in the Thoroughbred scan. The proportion of size variation explained was estimated using a normal linear model and by comparing the residual variance of a null model with sex only (V_N_) to a full model (V_F_) with sex and relevant markers. The proportion of explained variance is defined as 1 - (V_F_/V_N_).

### Haplotype Inference

Haploview [36] was used to assess patterns of linkage disequilibrium at the *LCORL* and *HMGA2* loci and blocks of contiguous SNPs were chosen for haplotype inference based on those patterns. Haplotypes were inferred with PHASE [37] using the default parameter values. Due to the small number of samples for each of the 16 breeds, the haplotype inference was conducted using the entire sample set together.
